# NBPT improves root morphology and yield of cotton under salinity through changes in root nitrogen-metabolizing enzymes

**DOI:** 10.3389/fpls.2026.1960114

**Published:** 2026-09-23

**Authors:** Wenbin Deng, Zhenfeng Miao, Haiping Xie, Xia Wang, Rongjiang Yao, Jinlin Chen, Guangming Liu

**Affiliations:** 1 Co-Innovation Center for Sustainable Forestry in Southern China, Nanjing Forestry University, Nanjing, China; 2 State Key Laboratory of Soil and Sustainable Agriculture, Institute of Soil Science, Chinese Academy of Sciences, Nanjing, China; 3 Department of Resources and Environment, Shandong Water Conservancy Vocational College, Rizhao, China

**Keywords:** cotton, NBPT, nitrogen use efficiency, root morphology, salinity

## Abstract

Rapid urea hydrolysis often limits nitrogen use efficiency (NUE) in cotton, especially under salinity. The urease inhibitor NBPT reduces ammonia loss, yet its effects on root morphology, nitrogen-metabolizing enzymes and exudation under salinity remain unclear. We conducted a greenhouse pot experiment, complemented by a two-year field trial, to test cotton responses to urea with or without NBPT under low (LS) and moderate (MS) salinity. NBPT increased total root length, surface area, volume and dry mass under both salinity levels. Root exudation of total organic carbon, organic acids, sugars and amino acids declined. At the same time, root urease activity fell, root urea content rose, activities of GS, GOGAT and AS increased, GDH activity decreased, and the GS/GDH ratio rose. These changes were accompanied by higher nitrogen uptake and by higher seed cotton yield and NUE in the pot experiment. Relative to urea alone, pot yield was 12.1% higher under LS and 20.5% higher under MS; the MS contrast was significant. A salinity × treatment interaction was detected for root urea content and pot NUE, but not for root morphology or yield. Redundancy analysis summarized the joint root-trait pattern. Across treatment means, larger roots and higher urea content co-occurred with higher pot yield. Field data followed the same direction for root dry mass, root urease activity, root urea content and seed cotton yield. NBPT therefore improved cotton performance under salinity through coordinated changes in root morphology and nitrogen-metabolizing enzyme activities.

## 1 Introduction

Soil salinization is a major limit on cotton production in arid and semi-arid regions. In Xinjiang, China’s largest cotton-growing area, saline and saline-alkali soils cover a large share of the cultivated land and keep expanding under irrigation and climate pressure (Dong et al., 2009; Dong, 2012; Muhammad et al., 2025). Salt stress restricts root elongation, reduces the proportion of fine roots and impairs nutrient and water uptake, ultimately lowering seed cotton yield and fiber quality (Zhang et al., 2014). Urea remains the main nitrogen fertilizer worldwide because of its high N content and low cost. Yet rapid hydrolysis by soil urease often causes ammonia volatilization, short-term ammonium toxicity and low nitrogen use efficiency (NUE), especially under the high pH or warm conditions common in cotton regions (Cantarella et al., 2018; Silva et al., 2017).

The urease inhibitor N-(n-butyl) thiophosphoric triamide (NBPT) slows urea hydrolysis, cuts ammonia loss by about 50% and raises crop yield and NUE by 5–13% on average (Abalos et al., 2014; Cantarella et al., 2018; Silva et al., 2017). Field and pot trials with maize, wheat and rice have shown that NBPT-treated urea improves plant N recovery and biomass relative to untreated urea (Dawar et al., 2011; Galindo et al., 2020; Zaman et al., 2009). Although NBPT is used mainly as a soil urease inhibitor, it can also act on roots. In hydroponic maize, NBPT reduced DUR3-mediated urea uptake and assimilation (Zanin et al., 2015). Those roots were exposed to NBPT in solution, with no soil and no salt. Whether cotton responds in the same way when urea and NBPT are applied to saline soil in split doses is not known.

Roots are the main interface for salt sensing and nitrogen uptake. The glutamine synthetase–glutamate synthase (GS-GOGAT) cycle is the primary pathway for ammonium assimilation under normal conditions and is central to efficient nitrogen use (Bernard and Habash, 2009; Lea and Miflin, 2011). By contrast, the glutamate dehydrogenase (GDH) pathway is often induced under high ammonium or abiotic stress and is usually viewed as a complementary or detoxifying route (Skopelitis et al., 2006; Tercé-Laforgue et al., 2004). The GS/GDH activity ratio was therefore used as a simple indicator of relative GS and GDH activities. Elevated ammonium restricts root cell expansion and lateral-root formation (Britto and Kronzucker, 2002; Hachiya and Sakakibara, 2017). Controlled urea supply, however, can ease ammonium-induced root growth inhibition in cotton through metabolic changes that involve arginine and polyamines (Liu et al., 2021). Root exudation of organic acids, sugars and amino acids also tends to rise under salt stress, contributing to osmotic adjustment and rhizosphere modification (Canarini et al., 2019; Sasse et al., 2018; Zhang et al., 2014).

Although NBPT has been widely evaluated for soil N dynamics and ammonia loss, evidence for its effects on cotton root urease, internal urea and the GS/GDH ratio under salinity is still limited. We therefore focused on the plant root side, using a greenhouse pot experiment and a two-year field trial. We hypothesized that soil-applied NBPT would lower root urease activity, raise root urea content and elevate the GS/GDH ratio under salinity, and that these changes would be accompanied by greater root growth and higher seed-cotton yield. The specific objectives were: (i) to quantify the effects of NBPT on root morphological traits, exudation rates, nitrogen-metabolizing enzyme activities and endogenous urea and ammonium contents under low and moderate salinity; (ii) to examine the multivariate relationships among these root traits; and (iii) to relate the root responses to agronomic performance and to test whether selected traits changed in the same direction in a two-year field trial.

## 2 Materials and methods

### 2.1 Experimental design and plant growth conditions

A two-factor completely randomized experiment was conducted in a greenhouse at Shihezi, Xinjiang, China. Day/night temperatures were kept at 25–30 °C/18–22 °C, relative humidity at 60–70%, and the photoperiod at 14 h d^-^¹ (light intensity ≥ 500 μmol m^-^² s^-^¹). Soil was collected from the 0–40 cm layer of two nearby saline cotton fields in Shihezi (Section 2.6). The fields already differed in total soluble salts. Salt was not added, and the two soils were not mixed. Low salinity (LS) was 3.63 ± 0.15 g kg^-^¹ (EC_1_:_5_ 0.90 ± 0.04 dS m^-^¹) and moderate salinity (MS) was 5.50 ± 0.18 g kg^-^¹ (EC_1_:_5_ 1.39 ± 0.06 dS m^-^¹). Other properties are given in **Supplementary Table S1A**.

Six treatments were set up: (1) LS-CK (no nitrogen), (2) LS-U (urea at 240 kg N ha^-^¹), (3) LS-UN (urea + NBPT), (4) MS-CK, (5) MS-U and (6) MS-UN. Thirty-three pots were prepared per treatment. The experimental unit was one pot with one plant. Of these, three pots were used only for root morphology at 72 h after the first fertilization and three only at 72 h after the ninth fertilization (**Supplementary Table S3**). At 24, 72, 120 and 168 h after the fifth fertilization, exudates and root morphology were measured on the same three pots; enzymes and root urea and ammonium were measured on three other pots. Three pots per treatment were kept until maturity for seed cotton yield and organ nitrogen uptake and were not sampled during the season. PVC columns (inner diameter 20 cm, height 60 cm) served as pots. A 5-cm layer of quartz sand (2–3 mm) was placed at the bottom as a filter. Air-dried, sieved soil was packed to 21.2 ± 0.2 kg per pot (bulk density 1.35 g cm^-^³; soil column height 50 cm).

Soil moisture was monitored with TDR sensors and held at 50–70% of field capacity (FC) during the seedling stage, 60–80% FC at the squaring stage, 65–85% FC at the flowering and boll-setting stage, and 55–75% FC at the boll-opening stage. Fertilizers (N-P_2_O_5_-K_2_O = 240-109–128 kg ha^-^¹; P as KH_2_PO_4_, K as K_2_SO_4_) and NBPT (1.0% of urea-N, w/w; purity ≥ 99%) were applied through a drip-irrigation system in nine splits at 7-day intervals (**Supplementary Table S2**). Seeds of cotton cultivar ‘Xinluzao 60’ were surface-sterilized and sown at three seeds per pot; seedlings were thinned to one plant at the cotyledon stage.

### 2.2 Root sampling and morphological analysis

Root samples were collected at 24, 72, 120 and 168 h after the fifth fertilization, at the flowering and boll-setting stage. Additional measurements were made at 72 h after the first fertilization (squaring stage) and at 72 h after the ninth fertilization (boll-opening stage); these data appear in **Supplementary Table S3**.

Intact root systems were recovered by carefully sawing the PVC columns open lengthwise. At 24, 72, 120 and 168 h after the fifth fertilization, exudates were collected first (Section 2.3). The same roots were then rinsed and scanned. Images were captured with an EPSON Expression 13000XL flatbed scanner and analyzed using WinRHIZO Pro software (Regent Instruments Inc., Canada). Total root length (cm), root surface area (cm²), average root diameter (mm), total root volume (cm³) and number of root tips were recorded. Roots were also sorted into diameter classes, and the proportions of fine roots (0–0.5 mm and 0.5–1.0 mm) were calculated. After scanning, roots were oven-dried at 70 °C to constant weight for determination of root dry mass (g plant^-^¹).

### 2.3 Collection and analysis of root exudates

Root exudates were collected from the same plants used for morphological analysis at 24, 72, 120 and 168 h after the fifth fertilization. After the columns were opened, intact roots were rinsed with deionized water and incubated in sterile deionized water for 2 h under greenhouse conditions. Roots were then scanned as described in Section 2.2. The trapping solution was passed through a 0.22 μm membrane filter and stored at once at −80 °C until analysis.

Total organic carbon (TOC) was measured with a TOC analyzer. Organic acids (citric, malic, oxalic, succinic, acetic and formic), sugars (glucose, fructose and sucrose) and amino acids (glutamic acid, aspartic acid, glutamine, asparagine, alanine, glycine, serine and threonine) were quantified by HPLC (Cawthray, 2003). Secretion rates were expressed on a root-dry-weight basis (μg C g^-^¹ root DW h^-^¹ for TOC; μmol g^-^¹ root DW h^-^¹ for organic acids, sugars and amino acids).

### 2.4 Determination of root enzyme activities and endogenous metabolites

Enzyme activities and root urea and ammonium contents were measured on a separate set of three pots per treatment at 24, 72, 120 and 168 h after the fifth fertilization.

Endogenous urease activity was measured by the indophenol method on fresh root extracts (Witte and Medina-Escobar, 2001) and expressed as mg urea g^-^¹ FW h^-^¹. Glutamine synthetase (GS) activity was determined by the hydroxamate biosynthetic assay (Lea et al., 1990). Glutamate synthase (GOGAT) and glutamate dehydrogenase (GDH) activities were measured spectrophotometrically by following NADH oxidation at 340 nm (Singh and Srivastava, 1986). Asparagine synthetase (AS) activity was assayed as described by Lea et al. (1990). Activities were expressed as μmol g^-^¹ FW h^-^¹ (μmol NADH g^-^¹ FW h^-^¹ for GDH). The GS/GDH ratio was calculated as an indicator of the relative activities of GS and GDH.

Root urea content was quantified by the diacetyl monoxime method (μg g^-^¹ FW). Ammonium (NH_4_^+^) content was determined by the indophenol blue method (mg kg^-^¹ FW).

### 2.5 Yield, nitrogen uptake and nitrogen use efficiency

At maturity, seed cotton was harvested from each treatment and weighed. Pot yield is reported as g plant^-^¹. Plants were divided into root, stem, leaf and boll, dried at 70 °C to constant weight and ground to pass a 0.15 mm sieve. Total nitrogen in each organ was measured by the Kjeldahl method. Organ N content (g N plant^-^¹) was calculated as organ dry mass × N concentration. Total plant N content was the sum of the four organs. NUE was calculated only for the pot experiment as seed cotton yield/total plant N content (g g^-^¹).

### 2.6 Field validation experiment

A two-year field trial was conducted in 2024 and 2025 at two nearby saline cotton fields in Shihezi, Xinjiang, China (approximately 44°18′ N, 86°05′ E; ca. 450 m a.s.l.). The climate is temperate continental arid, with a mean annual temperature of about 10 °C, a frost-free period of about 168 d, mean annual precipitation of about 193 mm and potential evaporation of about 1800 mm. Basic soil properties (0–40 cm) were measured before sowing (**Supplementary Table S1B**). The LS and MS fields were the source of the pot soils.

Cotton cultivar ‘Xinluzao 60’ was grown under plastic-mulched drip irrigation. Each mulch strip (2.05 m wide) carried six rows and three drip tapes (row spacing 10 + 66 + 10 + 66 + 10 cm), with 10 cm between plants and 66 cm between adjacent mulches. Fertilizer types and rates, and the NBPT rate, were the same as in the pot experiment.

Within each field, the three nitrogen treatments (CK, U and UN) were assigned at random to three plots. Plots were not grouped into blocks. Plot area was about 102 m² (four full mulch strips); one full mulch strip served as a guard between plots. Fertilizers and NBPT were applied in split doses through the drip system on the same schedule as in the pot experiment (**Supplementary Table S2**). The 2024 assignment was kept on the same plots in 2025, so the two years share the same experimental units.

Roots were sampled from the 0–40 cm layer with an auger at 72 h after the fifth fertilization, at the flowering and boll-setting stage. Root dry mass (g plant^-^¹ after conversion from sampling area and planting density), root urease activity and root urea content were determined as in Section 2.4. Seed cotton was harvested by plot at maturity and converted to yield per hectare (kg ha^-^¹). Partial factor productivity of N (PFP) was calculated as yield/N rate. Agronomic efficiency of N (AE) was calculated as (yield of the fertilized treatment − yield of CK in the same field)/N rate. PFP and AE were calculated only for U and UN.

### 2.7 Statistical analysis

Pot data were analyzed by three-way ANOVA with salinity, treatment and time as fixed factors in SPSS 26.0 or R version 4.5.2 (R Core Team, 2025). Residuals were checked for normality and homogeneity of variance (Shapiro–Wilk and Levene tests). Multiple comparisons were made with Tukey’s HSD test (*P* < 0.05). Results are given as mean ± SD (n = 3 independent pots per treatment at each pot sampling; n = 3 plots per year for field data). Significance is shown as *P* < 0.05, *P* < 0.01 and *P* < 0.001.

Field data (yield, PFP, AE, root dry mass, root urease activity and root urea content) were analyzed by ANOVA with year, field (LS, MS) and treatment as fixed factors. Year was treated as a fixed factor because the same plots were used in both years. Within each year and field, treatment means were compared using Tukey’s HSD test (*P* < 0.05). Salinity × treatment *P* values for the main pot and field traits are given in **Supplementary Table S7**.

Redundancy analysis (RDA) was performed with the vegan package in R after standardizing response variables. In the pot experiment, root traits and yield were measured on different plants. Pearson correlations with yield or NUE were therefore calculated from the six treatment × salinity means, using root variables averaged over 24–168 h. All figures were drawn with ggplot2 in R.

## 3 Results

### 3.1 Root morphology and dry mass

Both salinity and NBPT significantly affected root morphological traits (**Figure 1**; **Supplementary Table S3**). At 72 h after the fifth fertilization, total root length, root surface area, root volume and root dry mass ranked UN > U > CK under both salinity levels (*P* < 0.05). Relative to U, UN raised root dry mass by 22.2% under LS and 28.7% under MS, and raised root surface area by 26.3% and 44.9%. The salinity × treatment interaction was not significant when only U and UN were compared (**Supplementary Table S7c**). Main morphological traits kept increasing from 24 h to 168 h after fertilization and retained the order UN > U > CK at every sampling time. Average root diameter and fine-root proportions (0–0.5 mm and 0.5–1.0 mm) responded only weakly to treatment. The same ranking was observed at 72 h after the first (squaring stage) and ninth (boll-opening stage) fertilizations (**Supplementary Table S3**).

**Figure 1:**
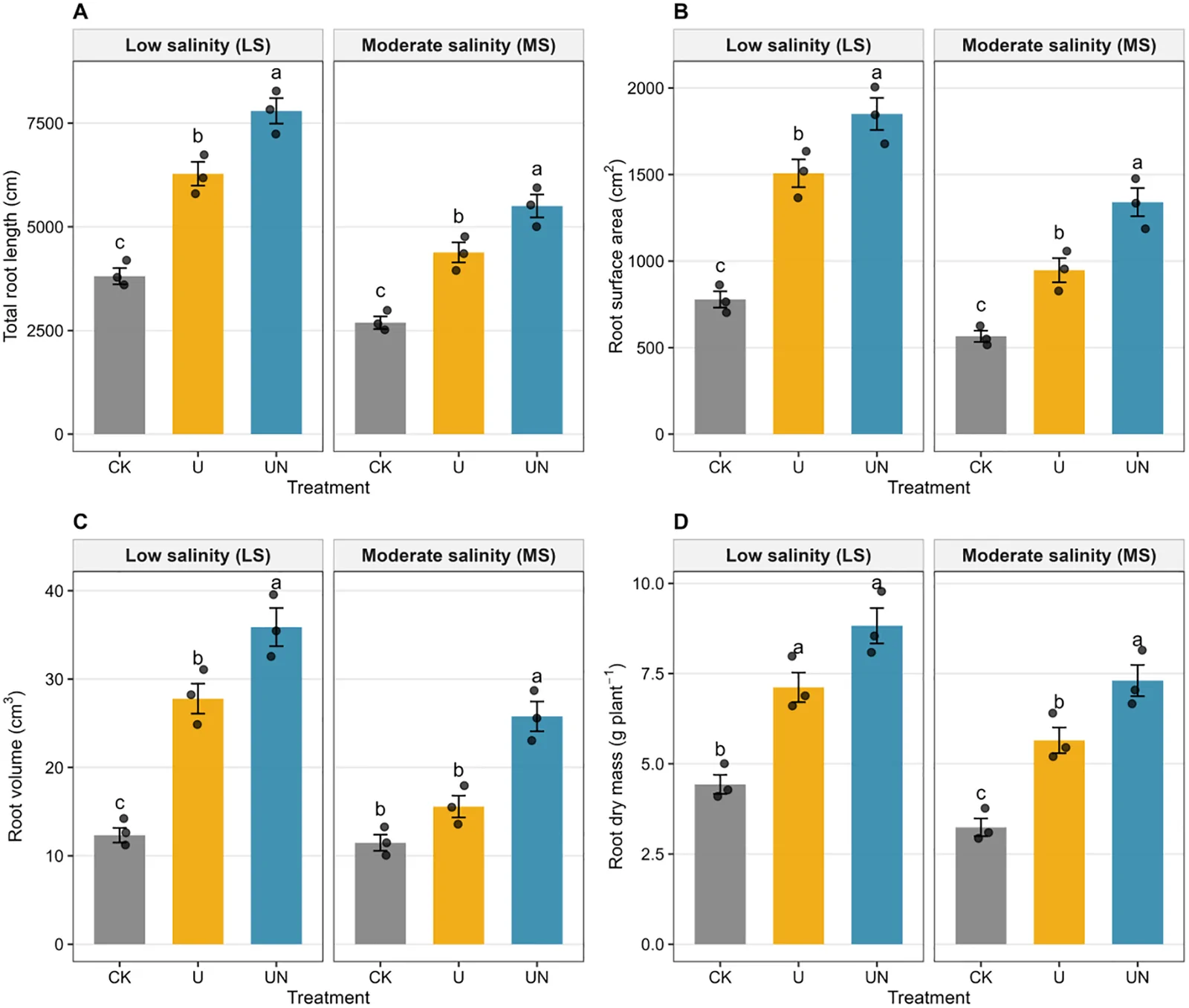
Effects of salinity and nitrogen treatment on cotton root morphology. **(A)** Total root length, **(B)** root surface area and **(C)** root volume at 72 h after the fifth fertilization (flowering and boll-setting stage). **(D)** Root dry mass at 24, 72, 120 and 168 h after the fifth fertilization. Different lowercase letters indicate significant differences among treatments within the same salinity (*P* < 0.05). Data are mean ± SD (n = 3). CK, no nitrogen; U, urea; UN, urea + NBPT.

### 3.2 Dynamics of root exudation

Both salinity and NBPT significantly affected root exudation rates (**Figure 2**). Total organic carbon (TOC), total organic acids, total sugars and total amino acids peaked at 24 h after fertilization and then declined. At each sampling time, exudation rates generally ranked U > UN > CK (*P* < 0.05). NBPT reduced exudation under both salinity levels. Relative to U, UN lowered the TOC exudation rate by 18–33% under low salinity and by 22–35% under moderate salinity. Total organic acids, total sugars and total amino acids showed similar declines. Moderate salinity raised overall exudation intensity, yet the relative reduction caused by NBPT was similar under the two salinity levels. Data for individual organic acids, sugars and amino acids are given in **Supplementary Table S4**.

**Figure 2:**
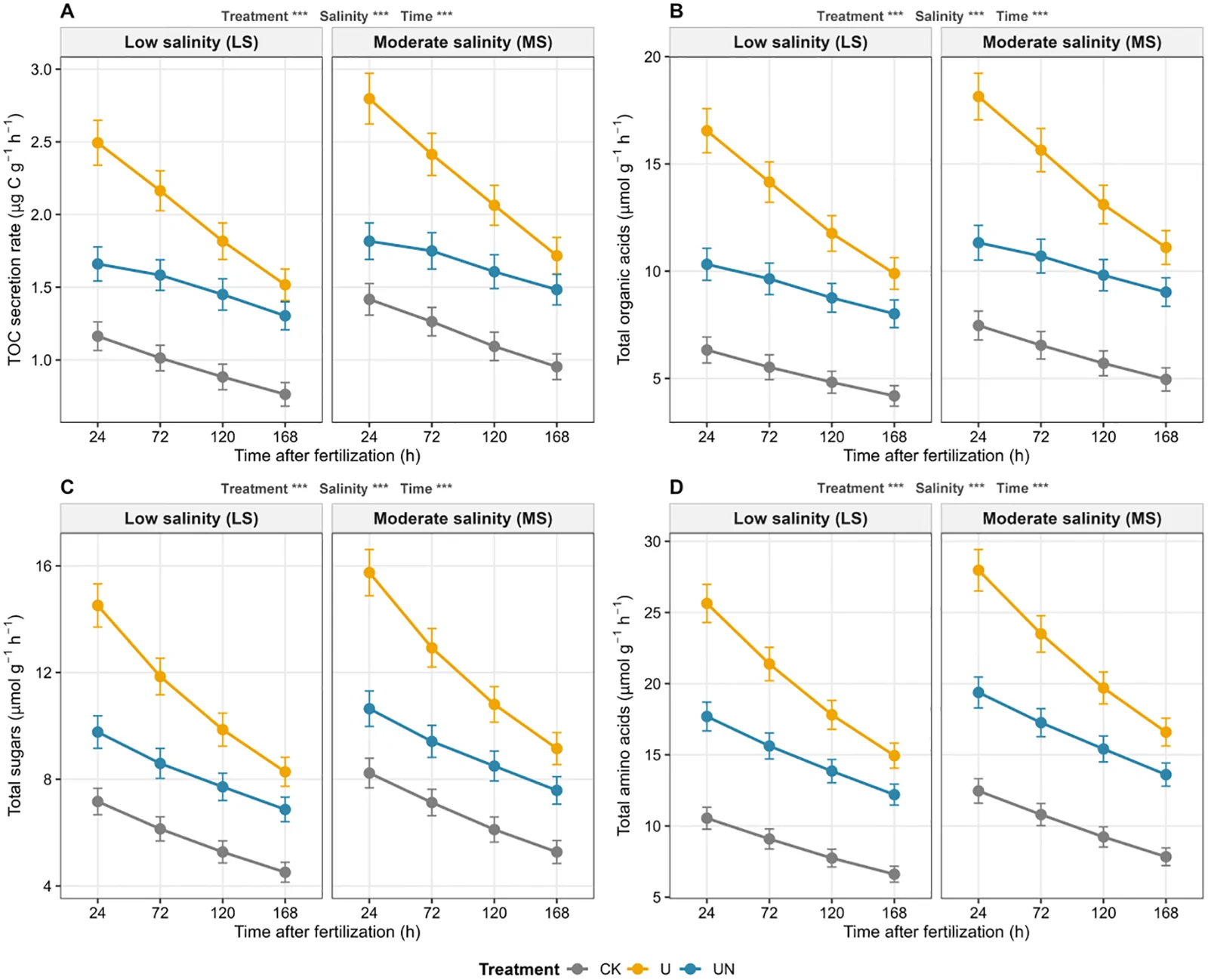
Dynamics of root exudation rates after the fifth fertilization. **(A)** Total organic carbon (TOC); **(B)** total organic acids; **(C)** total sugars; **(D)** total amino acids. Data are mean ± SD (n = 3). CK, no nitrogen; U, urea; UN, urea + NBPT. LS, low salinity; MS, moderate salinity. Asterisks in each panel indicate significant main effects of treatment, salinity and time (***P < 0.001); the salinity × treatment interaction was not significant.

### 3.3 Root enzyme activities, urea and ammonium contents

Both salinity and NBPT significantly affected root nitrogen-metabolizing enzyme activities and the contents of urea and ammonium (**Figure 3**). Urease activity ranked U > UN > CK under both salinity levels and declined steadily from 24 h to 168 h (*P* < 0.001). In contrast, GS, GOGAT and AS activities were highest under UN, intermediate under U and lowest under CK, while GDH activity showed the reverse order (U > UN > CK). The GS/GDH ratio was correspondingly higher under UN than under U and CK (**Supplementary Figure S1**). Root urea content was higher under UN than under U and CK, especially at 24–72 h. A salinity × treatment interaction was detected in the three-way ANOVA that included time (**Figure 3F**). Root NH_4_^+^ content was higher under U than under UN in the early period after fertilization (24–72 h), and the gap narrowed later.

**Figure 3:**
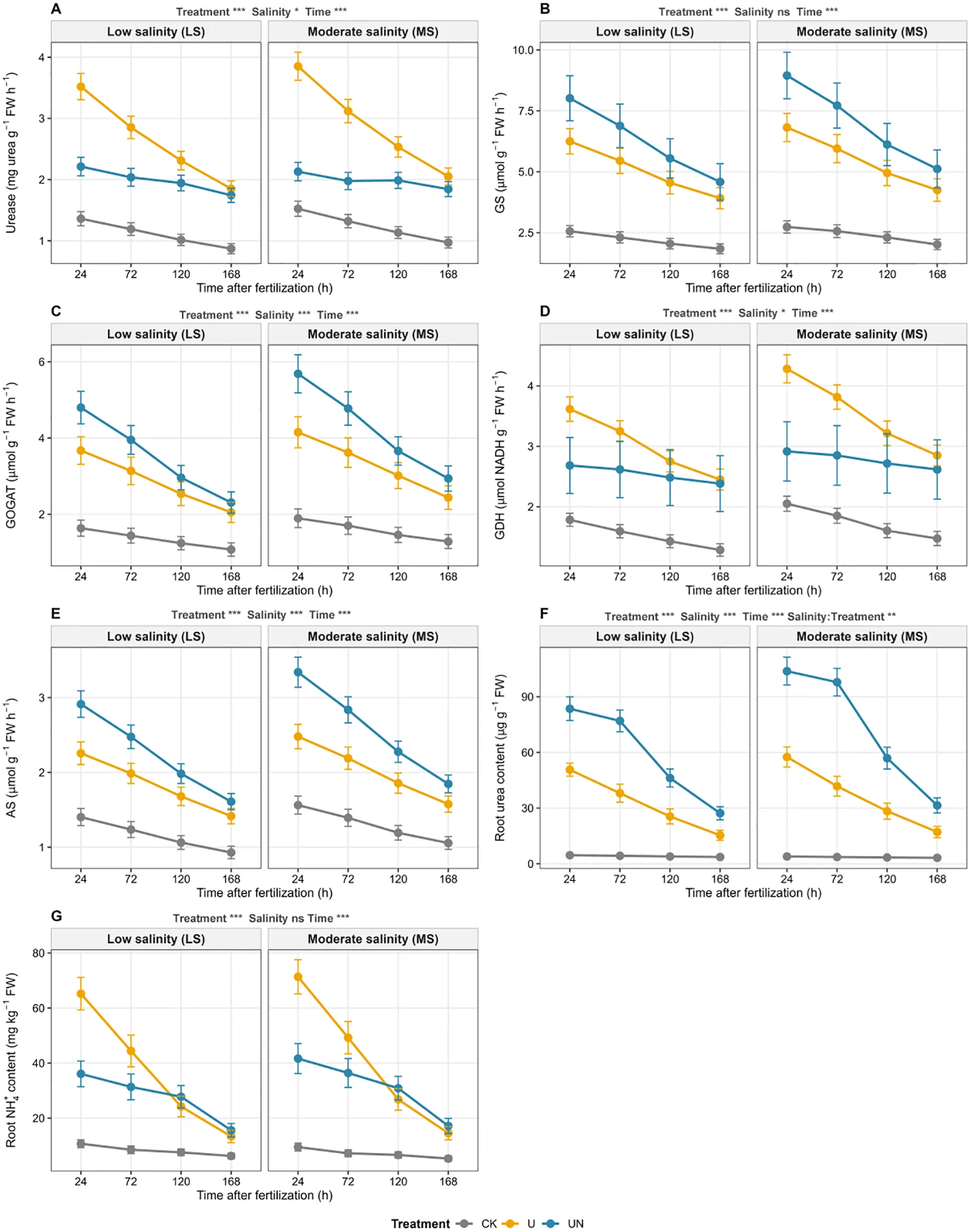
Dynamics of root nitrogen-metabolizing enzyme activities and endogenous metabolites after the fifth fertilization. **(A)** Urease; **(B)** glutamine synthetase (GS); **(C)** glutamate synthase (GOGAT); **(D)** glutamate dehydrogenase (GDH); **(E)** asparagine synthetase (AS); **(F)** root urea content; **(G)** root ammonium (NH_4_^+^) content. Data are mean ± SD (n = 3). CK, no nitrogen; U, urea; UN, urea + NBPT. LS, low salinity; MS, moderate salinity. Asterisks in each panel indicate significant effects of treatment, salinity and time (*P < 0.05, **P < 0.01, ***P < 0.001; ns, not significant). A significant salinity × treatment interaction was detected only for root urea content **(F)**.

### 3.4 Redundancy analysis of root morphological and physiological traits

Redundancy analysis (RDA) was run with salinity, treatment and time as constraining factors to explore links among root morphological traits, enzyme activities, urea and ammonium contents, and TOC exudation rate (**Figure 4**; **Supplementary Table S5**). The constrained model explained 85.7% of the total variance (adjusted *R*² = 0.844; *P* = 0.001). RDA1 and RDA2 accounted for 54.1% and 21.4% of the total variance. Variance partitioning attributed the largest unique fraction to treatment (adjusted *R*² = 0.674), then to time (0.167) and salinity (0.052) (**Supplementary Table S5**). RDA1 mainly separated CK from U and UN. On RDA2, U aligned with urease, GDH, TOC exudation and root NH_4_^+^, and UN with root urea, the GS/GDH ratio, root dry mass and root surface area.

**Figure 4:**
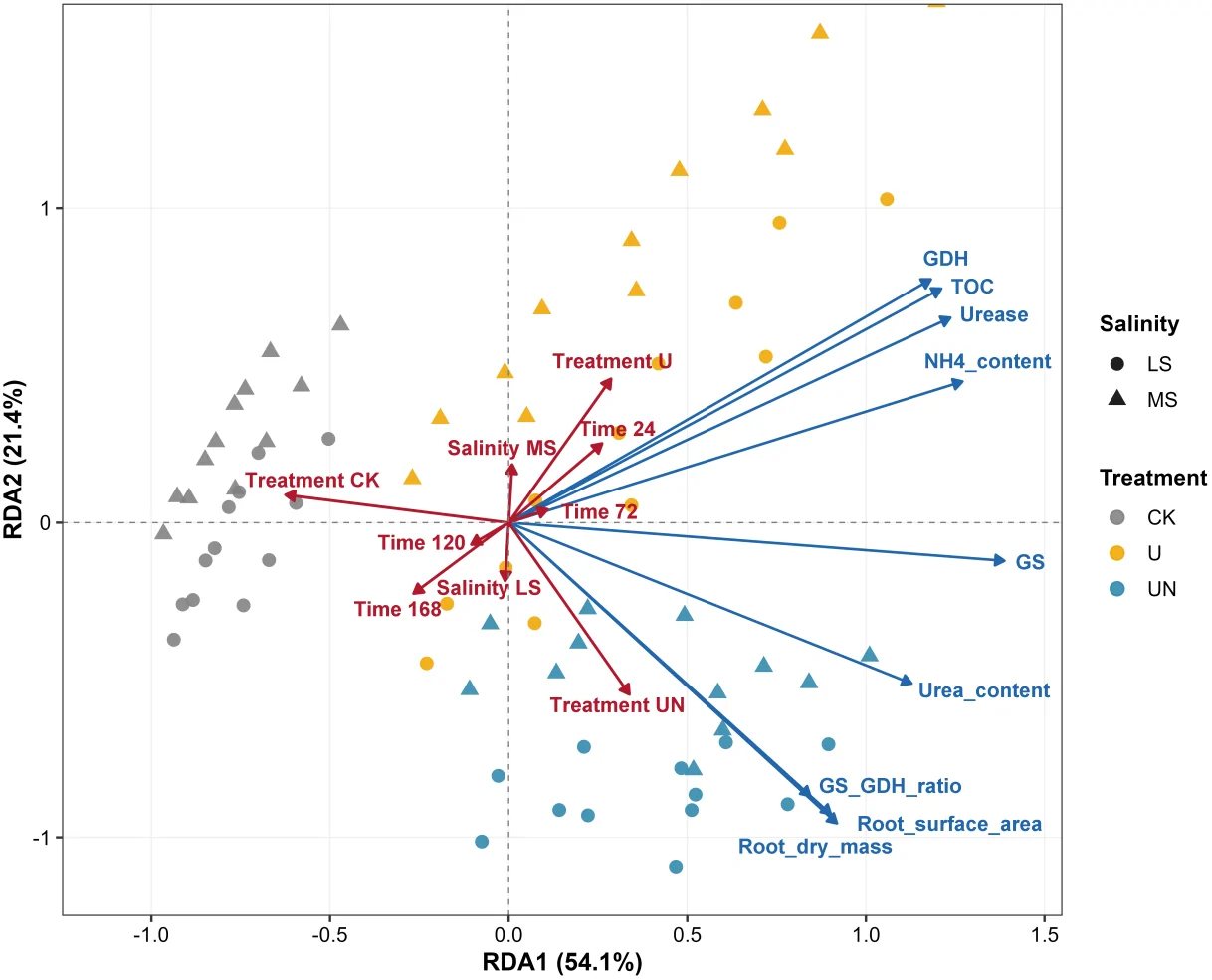
Redundancy analysis (RDA) of root traits constrained by salinity, treatment and time. Points are samples (color, treatment; shape, salinity). Blue arrows are response variables; red arrows are centroids of the constraining factors. Axis labels give the share of total variance.

### 3.5 Seed cotton yield, plant N content and nitrogen use efficiency

Seed cotton yield, organ N content and NUE were affected by salinity and nitrogen treatment (**Figure 5**). Under low salinity, yield did not differ between UN and U, and both exceeded CK. Under moderate salinity, yield ranked UN > U > CK (*P* < 0.05). Relative to U, yield under UN was 12.1% higher under LS (not significant) and 20.5% higher under MS. The salinity × treatment interaction for yield was significant when CK was included, but not when the analysis was restricted to U and UN (**Supplementary Table S7**). Plant N content was higher under U and UN than under CK, mainly in bolls and leaves. NUE ranked CK > UN > U under both salinity levels (*P* < 0.05), and the salinity × treatment interaction for NUE was significant (**Supplementary Table S7**). Across the six treatment means, yield increased with root dry mass and root surface area, and NUE declined as root urease activity and root NH_4_^+^ content increased (**Supplementary Table S6**).

**Figure 5:**
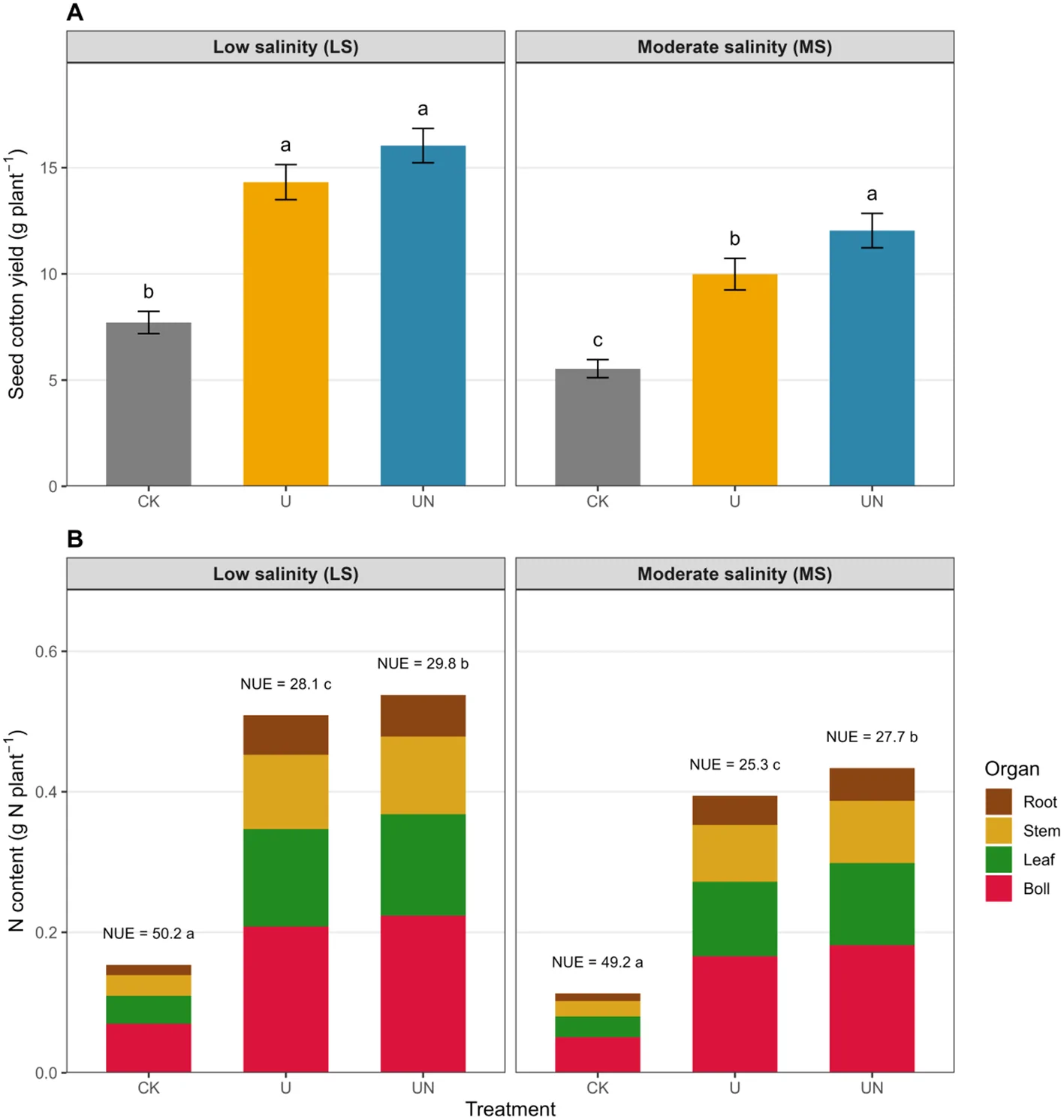
Seed cotton yield, organ N content and nitrogen use efficiency (NUE) at harvest in the pot experiment. **(A)** Seed cotton yield (g plant^-^¹). **(B)** Organ N content (g N plant^-^¹). Numbers above the bars are NUE (g g^-^¹); letters refer to NUE in **(B)** and to yield in **(A)**. Different letters indicate significant differences among treatments within the same salinity (Tukey’s HSD, *P* < 0.05). Data are mean ± SD (n = 3). CK, no nitrogen; U, urea; UN, urea + NBPT. LS, low salinity; MS, moderate salinity.

### 3.6 Field validation

Relative to U, UN increased seed cotton yield by about 7% under low salinity and about 10% under MS (**Figure 6A**). PFP and AE followed the same pattern, with a clearer numerical advantage under moderate salinity (**Figures 6B, C**). Treatment effects of U versus UN were significant for yield, root dry mass, root urease activity and root urea content; the salinity × treatment interaction was not significant for these traits (**Supplementary Table S7**).

**Figure 6:**
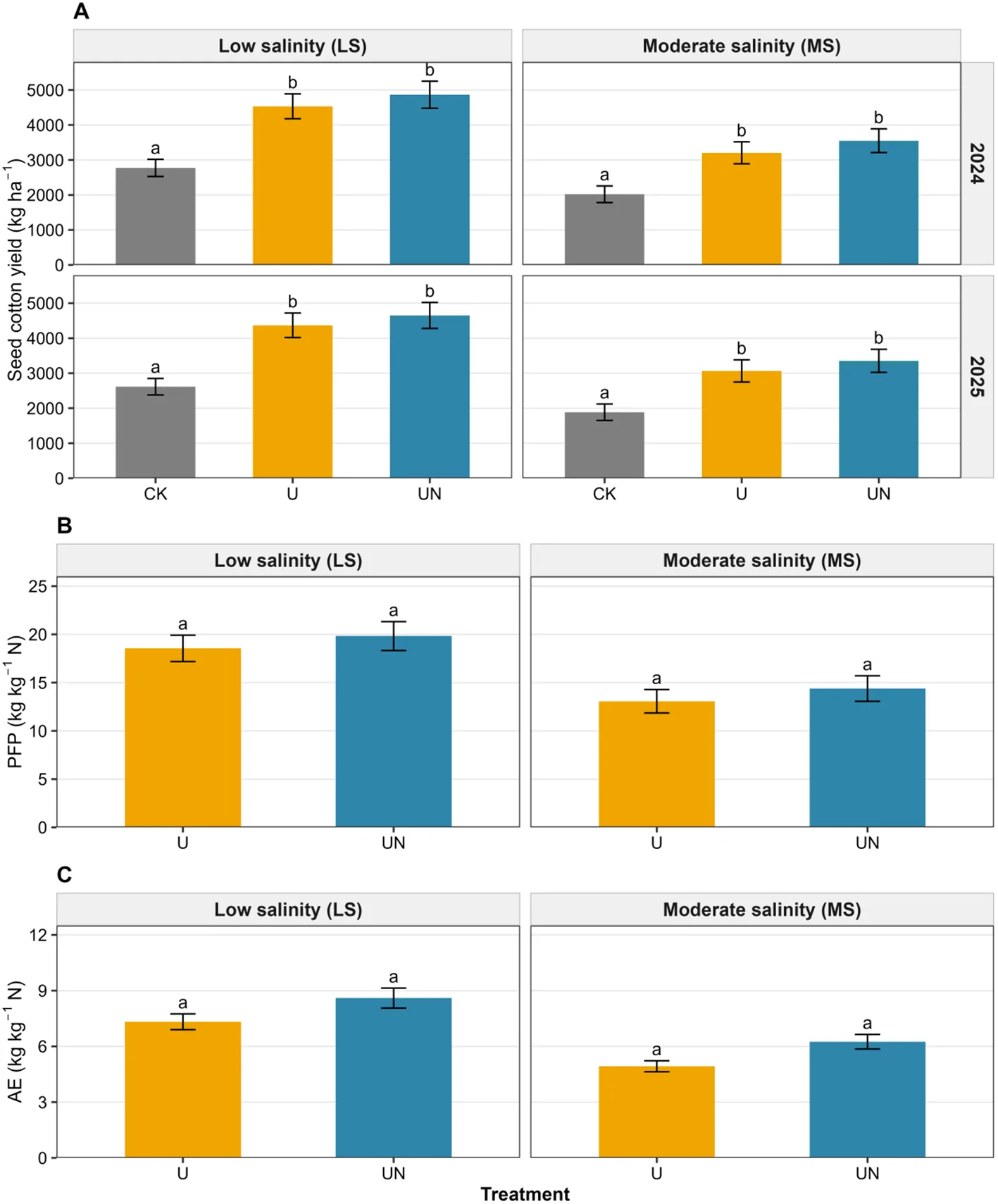
Field validation of seed cotton yield, partial factor productivity of nitrogen (PFP) and agronomic efficiency of nitrogen (AE) in 2024 and 2025. **(A)** Seed cotton yield. **(B)** PFP. **(C)** AE. Panels are separated by year and salinity level (LS, low salinity; MS, moderate salinity). Different letters indicate significant differences among treatments within the same salinity and year (Tukey’s HSD, P < 0.05). Data are mean ± SD (n = 3). CK, no nitrogen; U, urea; UN, urea + NBPT. PFP and AE were calculated only for the U and UN treatments.

At 72 h after the fifth fertilization, root dry mass was higher under UN than under U, and the difference was larger under moderate salinity (**Figure 7A**). Root urease activity ranked U > UN > CK (*P* < 0.05; **Figure 7B**). UN lowered urease activity by approximately 20% under LS and 26–28% under MS. Root urea content ranked UN > U > CK (*P* < 0.05; **Figure 7C**) and increased more under moderate salinity. These field patterns of root dry mass, urease activity and urea content were directionally consistent with the pot experiment and were larger under moderate salinity.

**Figure 7:**
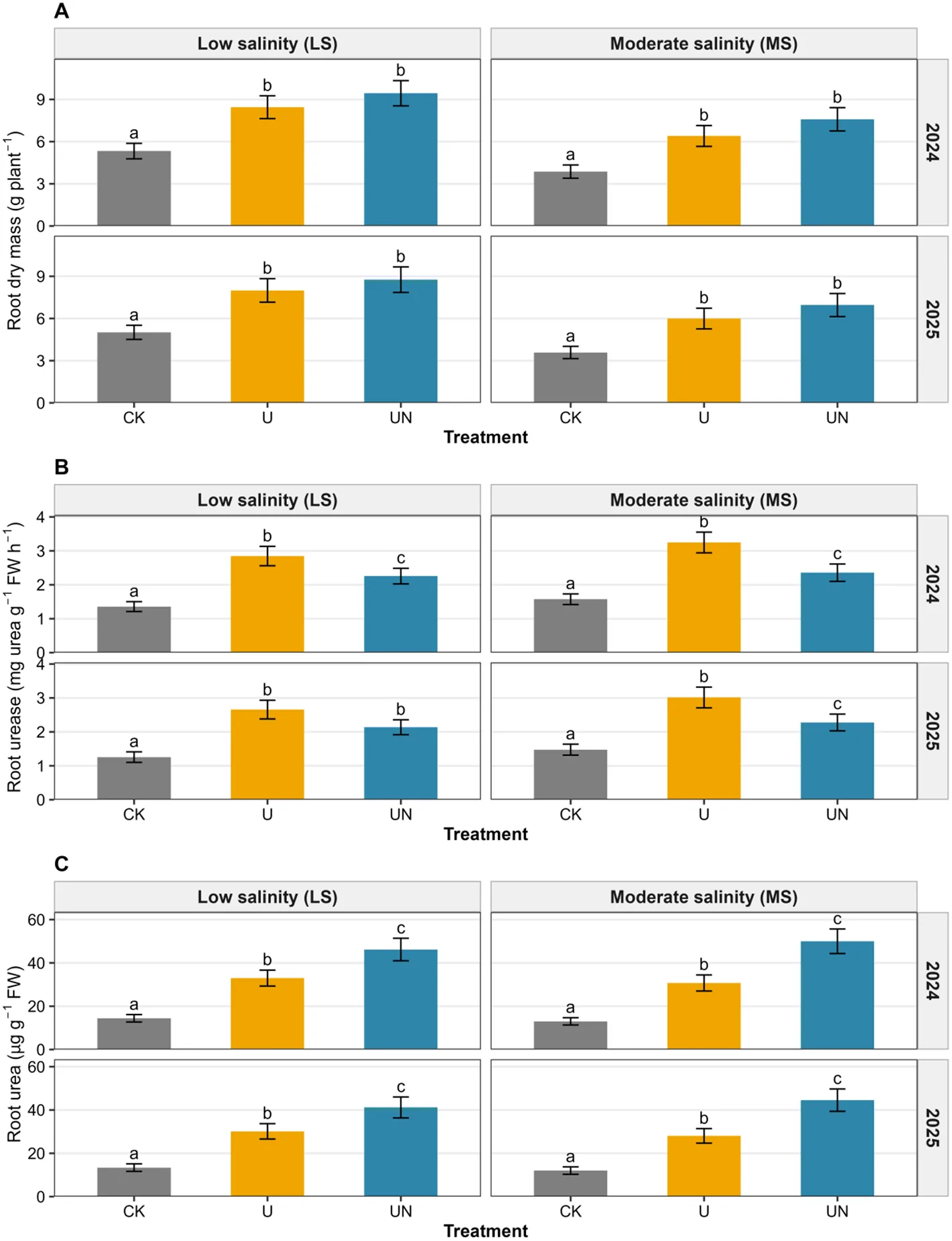
Field validation of root dry mass, root urease activity and root urea content at 72 h after the fifth fertilization in 2024 and 2025. **(A)** Root dry mass. **(B)** Root urease activity. **(C)** Root urea content. Panels are separated by year and salinity level (LS, low salinity; MS, moderate salinity). Different letters indicate significant differences among treatments within the same salinity and year (Tukey’s HSD, *P* < 0.05). Data are mean ± SD (n = 3). CK, no nitrogen; U, urea; UN, urea + NBPT.

## 4 Discussion

### 4.1 NBPT improves root morphology under low and moderate salinity

NBPT promoted cotton root growth under both salinity levels (**Figure 1**). Relative increases in root dry mass and surface area were larger under MS than under LS, but the salinity × treatment interaction was not significant for these traits when U and UN were compared (**Supplementary Table S7**). Salt stress commonly reduces cotton root length, surface area and dry mass (Zhang et al., 2014). The UN ranking held from 24 to 168 h after the fifth fertilization and at the first and ninth fertilizations (**Supplementary Table S3**), so the morphological response was not confined to a single sampling time. Field root dry mass showed the same treatment ranking (**Figure 7A**) and is discussed with yield in Section 4.5.

High ammonium limits root cell expansion and lateral-root formation (Britto and Kronzucker, 2002; Hachiya and Sakakibara, 2017), while exogenous urea can ease ammonium-induced root inhibition in cotton through the arginine–polyamine pathway (Liu et al., 2021). The higher root urea under UN (Section 4.3) is consistent with the recovery of root morphology.

Soil processes may also have played a part. By slowing urea hydrolysis, NBPT can blunt the early rhizosphere ammonium peak and lower the risk of local ammonium toxicity to roots (Cantarella et al., 2018; Dawar et al., 2011; Zaman et al., 2009). Yet root dry mass and surface area co-varied with root urea, the GS/GDH ratio and root ammonium in the same plants (**Figure 3**; the same pattern is summarized in **Figure 4**). Overall, the morphological data show that NBPT improved root growth under both salinity levels.

### 4.2 NBPT reduces the intensity of root exudation

Compared with urea alone, NBPT consistently lowered the exudation rates of total organic carbon, organic acids, sugars and amino acids (**Figure 2**). Exudation peaked soon after fertilization and then fell under all treatments. Individual compounds followed the same ranking (**Supplementary Table S4**).

Higher root exudation is a common response to nutrient imbalance or abiotic stress. Plants release organic acids, sugars and amino acids to mobilize nutrients, keep osmotic balance or attract beneficial microbes (Canarini et al., 2019; Sasse et al., 2018; Vives-Peris et al., 2020). Under salt stress, cotton and other crops often increase the release of organic acids and soluble sugars as part of osmotic adjustment and ion detoxification (Zhang et al., 2014). Against this background, the lower rates under UN suggest a smaller demand for exudate release in the trapping assay. The rates are comparative values among treatments, not *in situ* rhizosphere fluxes (Section 4.6).

When internal nitrogen supply is adequate, plants often release less sugar, amino acid and organic acid in trapping assays (Carvalhais et al., 2011; Zeng et al., 2024). In maize, nitrogen deficiency raised these fluxes, whereas ample nitrogen suppressed them (Carvalhais et al., 2011). The same treatment ranking was seen for exudation as for the enzyme and urea traits in Section 4.3, which argues against a direct effect of NBPT on membrane permeability.

Moderate salinity raised overall exudation intensity, yet the relative reduction caused by NBPT was similar under both salinity levels.

### 4.3 NBPT alters root nitrogen-metabolizing enzyme activities and the GS/GDH ratio

Under UN, root urease activity was lower than under U, and root urea content was higher, particularly under moderate salinity (**Figure 3**). At the same time, GS, GOGAT and AS activities increased, GDH activity fell, and the GS/GDH ratio rose (**Supplementary Figure S1**). The same directional changes in root urease activity and urea content were seen in the two-year field trial (**Figures 7B, C**).

These changes match a shift in the relative activities of GS and GDH. In higher plants, the GS-GOGAT cycle is the main route for incorporating ammonium into amino acids under normal conditions (Bernard and Habash, 2009; Lea and Miflin, 2011). The GDH pathway is often induced under high ammonium or stress and is usually treated as a complementary or detoxifying route (Skopelitis et al., 2006; Tercé-Laforgue et al., 2004). The higher GS/GDH ratio under UN shows that GS activity increased relative to GDH. Root urea can be hydrolyzed gradually, and the parallel drop in root ammonium lowers the risk of ammonium toxicity (Britto and Kronzucker, 2002; Esteban et al., 2016; Hachiya and Sakakibara, 2017).

Similar patterns have been reported when urea supply is controlled or ammonium stress is eased. In cotton, exogenous urea can reduce ammonium-induced root growth inhibition through metabolic changes involving arginine and polyamines (Liu et al., 2021). In hydroponic maize, NBPT reduced urea uptake and assimilation (Zanin et al., 2015). Soil-grown cotton in this study did the opposite under UN: root urea increased, and GS and GOGAT activities were higher. Urea and NBPT were applied to soil in split doses, not to roots in solution. DUR3 was not measured, so the contrast is limited to enzyme activities and metabolite contents.

A salinity × treatment interaction for root urea content was detected in the three-way ANOVA that included time (**Figure 3F**). Urea accumulation under UN was numerically greater under moderate salinity. The same interaction was not significant for root morphology or yield (**Supplementary Table S7**).

NBPT first inhibits soil urease and can therefore slow the early rise in soil ammonium after urea application. Such a change in external N supply is consistent with the higher root urea and lower root NH_4_^+^ under UN. This paper focuses on plant root responses; the relative contributions of soil and root processes remain to be resolved.

NBPT lowered root urease activity, raised root urea and elevated the GS/GDH ratio, and thereby changed the balance of GS and GDH activities under both salinity levels.

### 4.4 Integrative evidence from multivariate and correlation analyses

Redundancy analysis summarizes the links among root traits measured on the same plants (**Figure 4**; **Supplementary Table S5**).

CK separated from U and UN on the first RDA axis, which mainly distinguished unfertilized plants from plants that received urea. On the second axis, UN was associated with root urea, the GS/GDH ratio, root dry mass and root surface area, and U with urease, GDH, TOC exudation and root NH_4_^+^. The pattern matches the single-trait results. It does not isolate an NBPT effect from the general response to N fertilizer. Joint changes in root N-assimilating enzymes and root morphology under different N supplies have been reported in other crops (Forde, 2014; Garnett et al., 2009; Xu et al., 2012).

Because root traits and yield were measured on different pot plants, Pearson coefficients were calculated from the six treatment × salinity means (**Supplementary Table S6**). Treatment means of root dry mass increased with yield, and treatment means of urease activity and root ammonium decreased with NUE. These coefficients compare treatments; they do not pair the root status of a plant with its own yield. Field plots showed the same ranking of root dry mass, urease, urea and yield (**Figures 6**, **7**).

Thus UN clustered with higher GS/GDH, root urea and root size, and U with higher urease, GDH and root ammonium.

### 4.5 Agronomic benefits: yield and nitrogen use efficiency

The agronomic outcome of the root-level changes was a rise in seed-cotton yield and NUE (**Figure 5**). In pots, yield under UN was 12.1% higher than under U at low salinity (not significant) and 20.5% higher at moderate salinity (*P* < 0.05). Field gains were smaller, about 7% and 10%. The salinity × treatment interaction for the U versus UN yield contrast was not significant (**Supplementary Table S7**). The pot NUE response did show an interaction.

Meta-analyses of NBPT-treated urea report average yield gains of about 5–11%, often larger under sub-optimal conditions (Abalos et al., 2014; Silva et al., 2017). The pot MS contrast sits above that range; the field contrasts sit within it.

The largest increases in plant N were in bolls and leaves, so extra N was allocated mainly to reproductive and photosynthetic tissues. Similar shifts have been reported when urea hydrolysis is slowed or ammonium stress is eased (Dawar et al., 2011; Zaman et al., 2009). NUE was higher under UN than under U at both salinity levels (*P* < 0.05; **Figure 5B**). CK remained highest because N uptake was much smaller. A higher NUE under UN than under U has been linked to improved root N metabolism and less luxury consumption (Garnett et al., 2009; Xu et al., 2012).

UN out-yielded U under moderate salinity in pots, and the field trial followed the same direction. NUE was higher under UN than under U at both salinity levels.

### 4.6 Limitations and future perspectives

Several limitations should be kept in mind. NBPT mainly inhibits soil urease and therefore changes the timing of urea hydrolysis and the supply of ammonium and nitrate in the rhizosphere. The root-level changes recorded here—urea accumulation, shifts in enzyme activities and morphological improvement—may therefore partly reflect altered external nitrogen supply. Soil urea, soil urease and soil inorganic N were not reported in this study, so soil-mediated and root-level effects cannot be separated.

The mechanistic work was done in a greenhouse pot system designed to mimic split-application drip fertigation. This allowed time-resolved root sampling, but it differs from continuous drip irrigation and from commercial saline cotton fields. A two-year field trial under drip irrigation showed the same direction for root dry mass, root urease activity, root urea content and seed cotton yield (**Figures 6**, **7**).

Field yield gains were smaller than in the pots, and roots were sampled only at 72 h after the fifth fertilization. Exudation was measured after a 2-h incubation of washed roots in deionized water. Transfer from soil to water can change the composition and amount of released compounds, so the rates should be read as comparative values among treatments, not as rhizosphere carbon loss *in situ*.

Future work can close the remaining gaps. Simultaneous measurement of soil and root nitrogen pools would help separate external supply effects from internal metabolic changes. Split-root or axenic culture systems could isolate direct root responses to NBPT or urea. Multi-site field trials with more frequent root sampling and full-plant nitrogen budgets would strengthen the agronomic evidence. Transcriptomic and metabolomic analyses would give molecular detail on the enzyme-activity changes under combined salt and nitrogen stress.

Despite these limits, the field trial showed the same treatment ranking for root dry mass, root urease, root urea and yield as the pot experiment.

## 5 Conclusion

NBPT improved root growth, nitrogen-metabolizing enzyme activities and agronomic performance of cotton under both low and moderate salinity. The inhibitor lowered root urease activity, raised root urea content, elevated the GS/GDH ratio, increased GS, GOGAT and AS activities, and reduced GDH activity. These changes were accompanied by better root morphology, lower exudation of carbon compounds, higher nitrogen uptake, and higher seed cotton yield and NUE in the pot experiment. A two-year field trial confirmed the same directional responses for root dry mass, root urease activity, root urea content and seed cotton yield. A salinity × treatment interaction was detected for pot NUE and, in the time-course ANOVA, for root urea content, but not for root morphology or yield. NBPT can therefore improve cotton performance in saline environments through coordinated changes in root morphology and nitrogen-metabolizing enzyme activities, providing a plant-side basis for more efficient urea use in salt-affected cotton systems under drip irrigation.

## Data Availability

The raw data supporting the conclusions of this article will be made available by the authors, without undue reservation.
