# Supplementary material for: NBPT improves root morphology and yield of cotton under salinity through changes in root nitrogen-metabolizing enzymes
Source: Front Plant Sci. 2026 Sep 23;17:1960114. doi: 10.3389/fpls.2026.1960114 (PMC13642804; doi:10.3389/fpls.2026.1960114)
Supplement: Supplementary file 1 [file DataSheet1.docx]

**Supplementary materials**

**NBPT improves root morphology and yield of cotton under salinity through changes in root nitrogen-metabolizing enzymes**

**Table S1A.** Basic physicochemical properties of the low- and moderate-salinity soils used in the pot experiment

| Test soil | Bulk Density  (g cm^-3^) | pH | Soil organic matter  (g kg^-1^) | Total Nitrogen  (g kg^-1^) | Total soluble salts (g kg^-1^) | EC_1:5_  (dS m⁻¹) |
| --- | --- | --- | --- | --- | --- | --- |
| Low salinity (LS) | 1.37±0.12 | 7.7±0.15 | 12.12±1.52 | 1.13±0.18 | 3.63±0.15 | 0.90±0.04 |
| Moderate salinity (MS) | 1.32±0.14 | 7.9±0.09 | 10.08±0.83 | 0.95±0.06 | 5.50±0.18 | 1.39±0.06 |

#### Note: Data are mean ± SD (n = 3). Soils were collected from the 0–40 cm layer of two nearby saline cotton fields in Shihezi. Salt was not added. EC₁:₅ was measured in a 1:5 (w/v) soil:water extract.

**Table S1B.** Basic physicochemical properties of the soils used in the field experiment

| Field | Bulk Density  (g cm^-3^) | pH | Soil organic matter  (g kg^-1^) | Total Nitrogen  (g kg^-1^) | Total soluble salts (g kg^-1^) | EC_1:5_  (dS m⁻¹) |
| --- | --- | --- | --- | --- | --- | --- |
| Low salinity (LS) | 1.35±0.08 | 7.7±0.18 | 12.87±1.22 | 1.18±0.15 | 3.60±0.21 | 0.87±0.06 |
| Moderate salinity (MS) | 1.31±0.07 | 7.9±0.15 | 10.54±0.93 | 0.97±0.12 | 5.53±0.28 | 1.45±0.08 |

Note: Data are mean ± SD (n = 3). Samples were collected from the 0–40 cm layer of the LS and MS fields before sowing. These fields provided the pot soils (Table S1A). EC₁:₅ was measured in a 1:5 (w/v) soil:water extract.

#### **Table S2**. Fertilization schedule and rates in the pot experiment

| Number of times | Period | Urea  (mg pot^-1^) | KH_2_PO_4_  (mg pot^-1^) | K_2_SO_4_  (mg pot^-1^) | NBPT  （mg pot^-1^） |
| --- | --- | --- | --- | --- | --- |
| 1 | Squaring stage | 94 | 47 | 0 | 0.43 |
| 2 | Squaring stage | 141 | 47 | 24 | 0.65 |
| 3 | Flowering and boll-setting stage | 236 | 94 | 24 | 1.09 |
| 4 | Flowering and boll-setting stage | 236 | 94 | 47 | 1.09 |
| 5 | Flowering and boll-setting stage | 236 | 94 | 47 | 1.09 |
| 6 | Flowering and boll-setting stage | 236 | 94 | 47 | 1.09 |
| 7 | Flowering and boll-setting stage | 236 | 94 | 47 | 1.09 |
| 8 | Flowering and boll-setting stage | 141 | 47 | 47 | 0.65 |
| 9 | Boll-opening stage | 94 | 47 | 47 | 0.43 |

Note: Fertilizers and NBPT were applied via a drip-irrigation system in nine split applications (every 7 d) to simulate local field practice. The total N, P₂O₅ and K₂O rates corresponded to 240, 109 and 128 kg ha⁻¹, respectively, calculated on the basis of the pot surface area. NBPT was applied at 1.0% (w/w) of the urea-N rate (purity ≥ 99%). The same N, P, K and NBPT rates and the same nine-split schedule were used in the field trial (Section 2.6). CK received no N fertilizer; U received urea alone; UN received urea + NBPT.

**Table S3.** Root morphological parameters of cotton at different growth stages and time points after fertilization

| Time | Salinity | Treatment | Total root length (cm) | Root surface area (cm²) | Average root diameter (mm) | Total root volume (cm³) | Number of root tips | Proportion of fine roots (0–0.5 mm) (%) | Proportion of fine roots (0.5–1.0 mm) (%) | Root dry mass  (g plant⁻¹) |
| --- | --- | --- | --- | --- | --- | --- | --- | --- | --- | --- |
| SS-72h | LS | CK | 1435 ± 147b | 276 ± 38ab | 0.52 ± 0.03a | 4.05 ± 0.57ab | 2646 ± 312b | 61.2 ± 4.1a | 25.2 ± 2.6a | 2.03 ± 0.25ab |
|  | LS | U | 2323 ± 195de | 452 ± 52cd | 0.56 ± 0.04a | 7.21 ± 0.79de | 3791 ± 370c | 55.2 ± 2.6a | 27.3 ± 1.5a | 3.41 ± 0.36de |
|  | LS | UN | 2682 ± 177f | 524 ± 45d | 0.54 ± 0.04a | 8.48 ± 0.87e | 3913 ± 345c | 56.7 ± 2.4a | 26.1 ± 1.1a | 3.94 ± 0.36e |
|  | MS | CK | 897 ± 140a | 172 ± 33a | 0.48 ± 0.03a | 2.48 ± 0.45a | 1702 ± 268a | 59.4 ± 4.0a | 25.8 ± 2.2a | 1.44 ± 0.22a |
|  | MS | U | 1559 ± 169bc | 301 ± 45b | 0.53 ± 0.03a | 4.74 ± 0.65bc | 2608 ± 306b | 53.5 ± 2.7a | 28.2 ± 1.5a | 2.48 ± 0.34bc |
|  | MS | UN | 1913 ± 174cd | 373 ± 49bc | 0.51 ± 0.04a | 6.02 ± 0.72cd | 2845 ± 309b | 55.2 ± 2.3a | 26.8 ± 1.5a | 2.98 ± 0.34cd |
| FBS-24h | LS | CK | 3808 ± 339ab | 778 ± 82ab | 0.64 ± 0.05a | 12.33 ± 1.43a | 7686 ± 677b | 59.1 ± 2.9a | 29.9 ± 1.9a | 4.43 ± 0.45ab |
|  | LS | U | 6280 ± 491d | 1507 ± 140c | 0.83 ± 0.09a | 27.78 ± 2.95b | 7205 ± 693ab | 52.7 ± 3.2a | 25.7 ± 1.9a | 7.12 ± 0.70cd |
|  | LS | UN | 7795 ± 532e | 1850 ± 161d | 0.73 ± 0.07a | 35.88 ± 3.76c | 7785 ± 610b | 56.4 ± 3.7a | 26.6 ± 2.3a | 8.83 ± 0.84d |
|  | MS | CK | 2687 ± 264a | 566 ± 57a | 0.64 ± 0.07a | 11.48 ± 1.58a | 5518 ± 483a | 58.3 ± 3.2a | 26.3 ± 2.0a | 3.24 ± 0.42a |
|  | MS | U | 4383 ± 422bc | 947 ± 121b | 0.73 ± 0.08a | 15.58 ± 2.13a | 5561 ± 607a | 56.0 ± 4.3a | 28.6 ± 2.7a | 5.65 ± 0.61bc |
|  | MS | UN | 5502 ± 479cd | 1341 ± 141c | 0.72 ± 0.08a | 25.78 ± 2.92b | 6518 ± 635ab | 56.6 ± 3.8a | 27.4 ± 2.3a | 7.31 ± 0.74cd |
| FBS-72h | LS | CK | 4264 ± 346b | 855 ± 86ab | 0.66 ± 0.05a | 14.53 ± 1.69a | 8342 ± 661bc | 55.8 ± 3.1a | 28.5 ± 2.4a | 5.11 ± 0.57ab |
|  | LS | U | 7120 ± 482c | 1704 ± 129c | 0.86 ± 0.07b | 32.88 ± 3.32b | 8139 ± 667bc | 49.9 ± 3.2a | 25.2 ± 2.1a | 8.41 ± 0.84cd |
|  | LS | UN | 8934 ± 572d | 2152 ± 157d | 0.75 ± 0.05ab | 42.40 ± 3.92c | 8742 ± 612c | 54.2 ± 2.7a | 25.6 ± 1.8a | 10.28 ± 1.04d |
|  | MS | CK | 2941 ± 297a | 620 ± 69a | 0.67 ± 0.05a | 12.64 ± 1.69a | 6095 ± 610a | 55.3 ± 3.4a | 25.5 ± 1.9a | 3.66 ± 0.46a |
|  | MS | U | 4933 ± 432b | 1041 ± 102b | 0.75 ± 0.06ab | 18.87 ± 2.09a | 6009 ± 548a | 52.7 ± 3.2a | 27.4 ± 2.3a | 6.58 ± 0.67bc |
|  | MS | UN | 6199 ± 469c | 1508 ± 132c | 0.75 ± 0.06ab | 31.19 ± 2.97b | 6752 ± 591ab | 53.0 ± 3.3a | 26.2 ± 2.0a | 8.47 ± 0.86cd |
| FBS-120h | LS | CK | 4600 ± 347b | 922 ± 84ab | 0.67 ± 0.05a | 16.12 ± 1.67ab | 9018 ± 632a | 53.4 ± 2.3a | 27.4 ± 1.9a | 5.60 ± 0.59ab |
|  | LS | U | 7697 ± 491d | 1842 ± 133c | 0.87 ± 0.07b | 36.21 ± 2.89c | 8775 ± 695a | 47.5 ± 2.7a | 24.1 ± 1.6a | 9.22 ± 0.86cd |
|  | LS | UN | 9263 ± 586e | 2211 ± 154d | 0.76 ± 0.05ab | 44.46 ± 3.85d | 9068 ± 645a | 51.7 ± 2.6a | 24.3 ± 1.8a | 11.02 ± 1.02d |
|  | MS | CK | 3100 ± 263a | 658 ± 60a | 0.68 ± 0.05a | 13.70 ± 1.52a | 6387 ± 540b | 53.1 ± 2.6a | 24.8 ± 1.6a | 4.08 ± 0.46a |
|  | MS | U | 5304 ± 364bc | 1134 ± 87b | 0.76 ± 0.06ab | 20.90 ± 1.85b | 6419 ± 475b | 50.4 ± 2.6a | 26.4 ± 1.9a | 7.28 ± 0.71bc |
|  | MS | UN | 6349 ± 435c | 1557 ± 125c | 0.76 ± 0.06ab | 32.43 ± 2.80c | 6852 ± 530b | 51.1 ± 2.6a | 24.9 ± 1.9a | 9.15 ± 0.91cd |
| FBS-168h | LS | CK | 4832 ± 373b | 969 ± 88ab | 0.69 ± 0.05a | 17.67 ± 1.89a | 9452 ± 745a | 51.9 ± 2.4a | 27.1 ± 2.0a | 6.00 ± 0.59ab |
|  | LS | U | 8155 ± 556d | 1950 ± 155c | 0.89 ± 0.07b | 39.86 ± 3.25b | 9250 ± 780a | 46.3 ± 3.1a | 24.0 ± 1.9a | 9.85 ± 0.91cd |
|  | LS | UN | 9729 ± 683e | 2432 ± 180d | 0.77 ± 0.05ab | 51.80 ± 4.80c | 9822 ± 830a | 50.4 ± 2.6a | 24.7 ± 1.9a | 11.68 ± 1.06d |
|  | MS | CK | 3276 ± 300a | 693 ± 70a | 0.70 ± 0.05a | 14.85 ± 1.80a | 6824 ± 565b | 51.2 ± 2.4a | 24.4 ± 2.0a | 4.40 ± 0.50a |
|  | MS | U | 5611 ± 405bc | 1232 ± 110b | 0.79 ± 0.06ab | 23.20 ± 2.35a | 6784 ± 565b | 49.4 ± 3.2a | 26.5 ± 2.2a | 7.82 ± 0.76bc |
|  | MS | UN | 6651 ± 485c | 1720 ± 145c | 0.78 ± 0.06ab | 37.79 ± 3.55b | 7318 ± 635b | 49.4 ± 2.5a | 24.8 ± 1.9a | 9.68 ± 0.91cd |
| BOS-72h | LS | CK | 5050 ± 359ab | 1017 ± 88ab | 0.69 ± 0.04a | 19.03 ± 1.79ab | 9807 ± 740a | 51.6 ± 2.1a | 28.4 ± 1.1b | 7.35 ± 0.72ab |
|  | LS | U | 8383 ± 535c | 1953 ± 145c | 0.88 ± 0.05b | 41.50 ± 3.10c | 9316 ± 630a | 47.5 ± 1.9a | 25.2 ± 1.2a | 12.02 ± 1.20cd |
|  | LS | UN | 9916 ± 640d | 2418 ± 170d | 0.78 ± 0.04ab | 53.40 ± 3.80d | 9552 ± 630a | 51.2 ± 2.0a | 24.9 ± 1.0a | 13.95 ± 1.35d |
|  | MS | CK | 3533 ± 280a | 763 ± 68a | 0.71 ± 0.04a | 16.22 ± 1.50a | 7352 ± 585b | 50.8 ± 2.6a | 25.4 ± 1.1ab | 5.27 ± 0.64a |
|  | MS | U | 5846 ± 401b | 1297 ± 110b | 0.80 ± 0.05ab | 25.13 ± 1.95b | 7150 ± 530b | 50.8 ± 2.0a | 27.5 ± 1.2ab | 9.25 ± 1.05bc |
|  | MS | UN | 6840 ± 450c | 1708 ± 130c | 0.79 ± 0.05ab | 40.13 ± 3.05c | 7651 ± 500b | 51.5 ± 1.9a | 25.8 ± 0.9ab | 10.92 ± 1.15c |

Note: Data are mean ± SD (n = 3). Different letters within the same time point indicate significant differences among treatments according to Tukey’s HSD test (P < 0.05). SS, squaring stage (after the first fertilization); FBS, flowering and boll-setting stage (after the fifth fertilization); BOS, boll-opening stage (after the ninth fertilization). LS, low salinity; MS, moderate salinity; CK, no nitrogen; U, urea; UN, urea + NBPT.

**Table S4.** Composition of root exudates at different time points after the fifth fertilization

(a) Total organic carbon (TOC) and total organic acids

| Time | Salinity | Treatment | Root TOC | Total organic acids (μmol g⁻¹ root DW h⁻¹) | Citric acid (μmol g⁻¹ root DW h⁻¹) | Malic acid (μmol g⁻¹ root DW h⁻¹) | Oxalic acid (μmol g⁻¹ root DW h⁻¹) | Succinic acid (μmol g⁻¹ root DW h⁻¹) | Acetic acid (μmol g⁻¹ root DW h⁻¹) | Formic acid (μmol g⁻¹ root DW h⁻¹) |
| --- | --- | --- | --- | --- | --- | --- | --- | --- | --- | --- |
| 24h | LS | CK | 1.16±0.17a | 6.33±1.05a | 1.22±0.19a | 1.32±0.19a | 1.13±0.17a | 1.03±0.17a | 0.93±0.17a | 0.71±0.15a |
|  | LS | U | 2.49±0.27c | 16.55±1.78c | 3.18±0.32c | 3.29±0.34c | 2.90±0.30c | 2.70±0.30c | 2.46±0.27c | 2.01±0.25c |
|  | LS | UN | 1.66±0.20ab | 10.32±1.29ab | 1.99±0.24b | 2.09±0.24b | 1.82±0.22ab | 1.68±0.22ab | 1.52±0.19ab | 1.22±0.19ab |
|  | MS | CK | 1.42±0.19ab | 7.47±1.16ab | 1.46±0.20ab | 1.52±0.22ab | 1.33±0.20ab | 1.22±0.19ab | 1.08±0.18ab | 0.86±0.17ab |
|  | MS | U | 2.80±0.30c | 18.14±1.88c | 3.49±0.34c | 3.59±0.37c | 3.16±0.34c | 2.96±0.30c | 2.72±0.29c | 2.21±0.25c |
|  | MS | UN | 1.82±0.22b | 11.33±1.41b | 2.17±0.25b | 2.26±0.27b | 1.99±0.24b | 1.86±0.24b | 1.68±0.22b | 1.35±0.19b |
| 72h | LS | CK | 1.01±0.15a | 5.52±1.00a | 1.08±0.18a | 1.16±0.18a | 0.99±0.17a | 0.89±0.17a | 0.78±0.15a | 0.61±0.15a |
|  | LS | U | 2.16±0.24cd | 14.15±1.63de | 2.73±0.30de | 2.80±0.30de | 2.47±0.28de | 2.33±0.27de | 2.11±0.25de | 1.72±0.22cd |
|  | LS | UN | 1.58±0.18b | 9.64±1.27bc | 1.89±0.24bc | 1.96±0.24bc | 1.68±0.22bc | 1.58±0.22bc | 1.42±0.19bc | 1.10±0.17ab |
|  | MS | CK | 1.26±0.17ab | 6.55±1.11ab | 1.30±0.20ab | 1.36±0.20ab | 1.17±0.19ab | 1.08±0.18ab | 0.93±0.17ab | 0.71±0.15a |
|  | MS | U | 2.41±0.25d | 15.64±1.75e | 3.00±0.30e | 3.08±0.32e | 2.73±0.30e | 2.56±0.30e | 2.36±0.27e | 1.91±0.25d |
|  | MS | UN | 1.75±0.22bc | 10.7±1.36cd | 2.09±0.24cd | 2.15±0.25cd | 1.86±0.24cd | 1.76±0.23cd | 1.58±0.22cd | 1.25±0.19bc |
| 120h | LS | CK | 0.88±0.15a | 4.82±0.89a | 0.96±0.17a | 1.01±0.15a | 0.85±0.15a | 0.78±0.15a | 0.69±0.14a | 0.52±0.12a |
|  | LS | U | 1.82±0.22cd | 11.76±1.44cd | 2.26±0.27cd | 2.33±0.27cd | 2.03±0.23cd | 1.93±0.23cd | 1.76±0.23cd | 1.46±0.20cd |
|  | LS | UN | 1.45±0.19bc | 8.76±1.16bc | 1.72±0.22bc | 1.75±0.22bc | 1.52±0.19bc | 1.45±0.19bc | 1.28±0.18bc | 1.04±0.17bc |
|  | MS | CK | 1.09±0.17ab | 5.71±1.00ab | 1.12±0.19ab | 1.18±0.18ab | 1.03±0.17ab | 0.96±0.17ab | 0.81±0.15ab | 0.60±0.14ab |
|  | MS | U | 2.06±0.24d | 13.11±1.56d | 2.52±0.29d | 2.58±0.28d | 2.26±0.27d | 2.16±0.27d | 1.96±0.24d | 1.62±0.22d |
|  | MS | UN | 1.61±0.20bcd | 9.81±1.26cd | 1.93±0.23cd | 1.96±0.24cd | 1.68±0.22c | 1.62±0.22cd | 1.45±0.19cd | 1.17±0.17cd |
| 168h | LS | CK | 0.76±0.14a | 4.19±0.83a | 0.81±0.15a | 0.88±0.15a | 0.76±0.14a | 0.69±0.14a | 0.58±0.12a | 0.45±0.12a |
|  | LS | U | 1.52±0.19c | 9.89±1.28c | 1.89±0.24c | 1.96±0.24cd | 1.72±0.22c | 1.62±0.22c | 1.48±0.18cd | 1.22±0.19cd |
|  | LS | UN | 1.30±0.17bc | 8.01±1.12bc | 1.56±0.20bc | 1.61±0.20bc | 1.42±0.19bc | 1.32±0.19bc | 1.17±0.17bc | 0.94±0.17bc |
|  | MS | CK | 0.95±0.15ab | 4.96±0.94ab | 0.99±0.17ab | 1.03±0.17ab | 0.89±0.17ab | 0.81±0.15ab | 0.71±0.15ab | 0.52±0.12ab |
|  | MS | U | 1.72±0.22c | 11.1±1.37c | 2.11±0.25c | 2.19±0.27d | 1.93±0.23c | 1.83±0.23c | 1.66±0.20d | 1.38±0.18d |
|  | MS | UN | 1.48±0.18c | 9.02±1.15c | 1.75±0.22c | 1.82±0.22cd | 1.58±0.18c | 1.48±0.18c | 1.32±0.19cd | 1.07±0.17cd |

(b) Total sugars and individual sugars (μmol g⁻¹ root DW h⁻¹)

| Time | Salinity | Treatment | Total sugar | Glucose | Fructose | Sucrose |
| --- | --- | --- | --- | --- | --- | --- |
| 24h | LS | CK | 7.17±0.86a | 2.41±0.29a | 2.26±0.27a | 2.50±0.30a |
|  | LS | U | 14.52±1.40a | 4.87±0.47c | 4.58±0.45c | 5.06±0.49c |
|  | LS | UN | 9.77±1.06ab | 3.28±0.35ab | 3.09±0.34ab | 3.39±0.37ab |
|  | MS | CK | 8.23±0.96ab | 2.77±0.32ab | 2.60±0.30ab | 2.86±0.34ab |
|  | MS | U | 15.75±1.50c | 5.27±0.50c | 4.98±0.48c | 5.49±0.52c |
|  | MS | UN | 10.64±1.15b | 3.58±0.38b | 3.36±0.37b | 3.70±0.40b |
| 72h | LS | CK | 6.14±0.78a | 2.07±0.25a | 1.94±0.25a | 2.13±0.27a |
|  | LS | U | 11.85±1.19cd | 3.95±0.39cd | 3.75±0.39cd | 4.15±0.42cd |
|  | LS | UN | 8.59±0.97ab | 2.88±0.32ab | 2.72±0.32ab | 2.99±0.34ab |
|  | MS | CK | 7.13±0.86ab | 2.41±0.29ab | 2.26±0.27ab | 2.46±0.30ab |
|  | MS | U | 12.93±1.25d | 4.32±0.42d | 4.11±0.40d | 4.50±0.44d |
|  | MS | UN | 9.42±1.04bc | 3.16±0.34bc | 2.99±0.34bc | 3.26±0.37bc |
| 120h | LS | CK | 5.28±0.72a | 1.76±0.24a | 1.67±0.22a | 1.84±0.25a |
|  | LS | U | 9.86±1.07cd | 3.28±0.35cd | 3.11±0.35cd | 3.46±0.37cd |
|  | LS | UN | 7.72±0.89abc | 2.58±0.28abc | 2.45±0.29abc | 2.68±0.32abc |
|  | MS | CK | 6.12±0.81ab | 2.06±0.27ab | 1.93±0.27ab | 2.13±0.27ab |
|  | MS | U | 10.81±1.16d | 3.58±0.38d | 3.43±0.37d | 3.80±0.40d |
|  | MS | UN | 8.5±0.97bcd | 2.82±0.32bcd | 2.72±0.32bcd | 2.96±0.34bcd |
| 168h | LS | CK | 4.52±0.65a | 1.52±0.22a | 1.43±0.20a | 1.57±0.22a |
|  | LS | U | 8.28±0.94c | 2.76±0.30c | 2.63±0.30c | 2.89±0.34c |
|  | LS | UN | 6.87±0.8bc | 2.30±0.27bc | 2.18±0.25bc | 2.38±0.28abc |
|  | MS | CK | 5.28±0.75ab | 1.77±0.25ab | 1.66±0.24ab | 1.84±0.25ab |
|  | MS | U | 9.15±1.04c | 3.06±0.34c | 2.89±0.34c | 3.19±0.37c |
|  | MS | UN | 7.58±0.89bc | 2.55±0.29c | 2.42±0.29c | 2.62±0.32bc |

(c) Total amino acids and individual amino acids (μmol g⁻¹ root DW h⁻¹)

| Time | Salinity | Treatment | Total amino acids | Glutamic acid | Aspartic acid | Glutamine | Asparagine | Alanine | Glycine | Serine | Threonine |
| --- | --- | --- | --- | --- | --- | --- | --- | --- | --- | --- | --- |
| 24h | LS | CK | 10.54±1.33a | 1.70±0.20a | 1.44±0.18a | 1.79±0.20a | 1.50±0.18a | 1.27±0.18a | 1.07±0.15a | 0.94±0.13a | 0.83±0.12a |
|  | LS | U | 25.64±2.32d | 3.79±0.31d | 3.38±0.30d | 4.16±0.35cd | 3.53±0.31cd | 3.06±0.30d | 2.76±0.27d | 2.57±0.25d | 2.38±0.24c |
|  | LS | UN | 17.69±1.75bc | 2.52±0.23bc | 2.24±0.22bc | 3.19±0.29b | 2.70±0.26b | 2.01±0.22bc | 1.81±0.19bc | 1.68±0.18bc | 1.54±0.17b |
|  | MS | CK | 12.46±1.5ab | 1.98±0.21ab | 1.70±0.20ab | 2.12±0.23a | 1.77±0.20a | 1.48±0.19ab | 1.28±0.17ab | 1.14±0.15ab | 0.98±0.13a |
|  | MS | U | 27.97±2.53d | 4.14±0.34d | 3.68±0.33d | 4.52±0.38d | 3.88±0.34d | 3.34±0.32d | 3.01±0.29d | 2.82±0.28d | 2.59±0.26c |
|  | MS | UN | 19.38±1.88c | 2.76±0.24c | 2.44±0.24c | 3.46±0.31bc | 2.96±0.29bc | 2.21±0.23c | 1.99±0.21c | 1.86±0.19c | 1.69±0.18b |
| 72h | LS | CK | 9.09±1.22a | 1.48±0.19a | 1.24±0.17a | 1.52±0.18a | 1.31±0.17a | 1.10±0.15a | 0.92±0.13a | 0.80±0.12a | 0.71±0.11a |
|  | LS | U | 21.38±2.04cd | 3.19±0.29d | 2.81±0.27d | 3.44±0.31bc | 2.95±0.28bc | 2.56±0.26d | 2.30±0.23d | 2.13±0.22c | 1.99±0.20c |
|  | LS | UN | 15.62±1.58b | 2.27±0.22bc | 1.97±0.21bc | 2.78±0.27b | 2.37±0.24b | 1.77±0.19bc | 1.59±0.17bc | 1.48±0.15b | 1.38±0.15b |
|  | MS | CK | 10.8±1.35a | 1.76±0.20ab | 1.48±0.19ab | 1.80±0.21a | 1.55±0.19a | 1.31±0.17ab | 1.10±0.14ab | 0.97±0.13a | 0.84±0.12a |
|  | MS | U | 23.5±2.22d | 3.48±0.31d | 3.09±0.29d | 3.78±0.34c | 3.25±0.31c | 2.81±0.27d | 2.53±0.25d | 2.36±0.24c | 2.19±0.22c |
|  | MS | UN | 17.26±1.7bc | 2.50±0.22c | 2.18±0.22c | 3.05±0.29bc | 2.62±0.26bc | 1.97±0.21c | 1.78±0.18c | 1.64±0.17b | 1.53±0.16b |
| 120h | LS | CK | 7.74±1.08a | 1.27±0.17a | 1.07±0.15a | 1.30±0.17a | 1.10±0.14a | 0.94±0.14a | 0.77±0.11a | 0.69±0.11a | 0.61±0.09a |
|  | LS | U | 17.81±1.77bc | 2.63±0.26de | 2.34±0.23de | 2.85±0.28bc | 2.45±0.25bc | 2.13±0.22de | 1.93±0.19cd | 1.80±0.18cd | 1.67±0.17cd |
|  | LS | UN | 13.85±1.42b | 2.00±0.20bc | 1.76±0.19bc | 2.41±0.24b | 2.07±0.21b | 1.60±0.17bc | 1.43±0.15b | 1.34±0.14b | 1.24±0.13b |
|  | MS | CK | 9.23±1.23a | 1.50±0.19ab | 1.28±0.17ab | 1.53±0.19a | 1.31±0.17a | 1.11±0.15ab | 0.94±0.13a | 0.84±0.12a | 0.72±0.11a |
|  | MS | U | 19.69±1.94c | 2.89±0.28e | 2.58±0.25e | 3.13±0.30c | 2.71±0.27c | 2.36±0.24e | 2.16±0.22d | 2.00±0.20d | 1.85±0.19d |
|  | MS | UN | 15.41±1.57b | 2.20±0.22cd | 1.96±0.21cd | 2.64±0.26bc | 2.31±0.23bc | 1.78±0.19cd | 1.61±0.17bc | 1.50±0.16bc | 1.40±0.15bc |
| 168h | LS | CK | 6.61±0.97a | 1.08±0.16a | 0.92±0.14a | 1.10±0.15a | 0.93±0.13a | 0.80±0.12a | 0.67±0.10a | 0.60±0.09a | 0.53±0.08a |
|  | LS | U | 14.94±1.52bc | 2.18±0.22cd | 1.97±0.21cd | 2.38±0.24b | 2.04±0.21bc | 1.80±0.18bc | 1.63±0.17bc | 1.51±0.16bc | 1.41±0.15bc |
|  | LS | UN | 12.2±1.28b | 1.76±0.19bc | 1.56±0.16bc | 2.10±0.21b | 1.76±0.18b | 1.43±0.15b | 1.28±0.14b | 1.20±0.13b | 1.11±0.12b |
|  | MS | CK | 7.84±1.08a | 1.28±0.17ab | 1.09±0.15ab | 1.30±0.17a | 1.10±0.14a | 0.94±0.13a | 0.80±0.12a | 0.71±0.11a | 0.62±0.09a |
|  | MS | U | 16.59±1.69c | 2.41±0.25d | 2.18±0.22d | 2.63±0.27b | 2.28±0.23c | 2.00±0.20c | 1.83±0.19c | 1.69±0.18c | 1.57±0.16c |
|  | MS | UN | 13.61±1.41bc | 1.95±0.21cd | 1.73±0.19cd | 2.31±0.23b | 1.97±0.20bc | 1.60±0.17bc | 1.44±0.15bc | 1.35±0.14bc | 1.25±0.13bc |

Note**:** Data are mean ± SD (n = 3). Different letters within the same time point indicate significant differences among treatments according to Tukey’s HSD test (P < 0.05). LS, low salinity; MS, moderate salinity; CK, no nitrogen; U, urea; UN, urea + NBPT.

**Table S5.** Redundancy analysis of root traits constrained by salinity, treatment and time

(a) Permutation tests (999 permutations)

| Source | df | Variance | *F* | *P* |
| --- | --- | --- | --- | --- |
| Model | 6 | 7.715 | 65.025 | 0.001 |
| Salinity | 1 | 0.452 | 22.838 | 0.001 |
| Treatment | 2 | 5.764 | 145.736 | 0.001 |
| Time | 3 | 1.500 | 25.279 | 0.001 |
| Residual | 65 | 1.285 | — | — |

(b) Eigenvalues

| Axis | Eigenvalue | Proportion of total variance | Cumulative |
| --- | --- | --- | --- |
| RDA1 | 4.87 | 0.541 | 0.541 |
| RDA2 | 1.927 | 0.214 | 0.755 |

(c) Response-variable scores (scaling = 2)

| Variable | RDA1 | RDA2 |
| --- | --- | --- |
| Root_dry_mass | 0.98 | -1.007 |
| Root_surface_area | 0.999 | -1.039 |
| Urease | 1.344 | 0.708 |
| GS | 1.509 | -0.132 |
| GDH | 1.285 | 0.841 |
| GS_GDH_ratio | 0.918 | -0.944 |
| Urea_content | 1.226 | -0.556 |
| NH4_content | 1.381 | 0.488 |
| TOC | 1.316 | 0.809 |

(d) Centroids of constraining factors (scaling = 2)

| Variable | RDA1 | RDA2 |
| --- | --- | --- |
| TreatmentCK | -0.762 | 0.108 |
| TreatmentU | 0.35 | 0.558 |
| TreatmentUN | 0.412 | -0.666 |
| SalinityLS | -0.012 | -0.226 |
| SalinityMS | 0.012 | 0.226 |
| Time24 | 0.319 | 0.307 |
| Time72 | 0.133 | 0.052 |
| Time120 | -0.127 | -0.085 |
| Time168 | -0.324 | -0.274 |

(e) Constrained model fit

| *R*^2^ | Adjusted *R*^2^ |
| --- | --- |
| 0.857 | 0.844 |

(f) Variance partitioning (unique fractions, adjusted *R*²)

| Fraction | Adjusted *R*^2^ |
| --- | --- |
| Salinity (controlling for treatment and time) | 0.052 |
| Treatment (controlling for salinity and time) | 0.674 |
| Time (controlling for salinity and treatment) | 0.167 |
| Residuals | 0.156 |

Note: Response variables were standardized before RDA. Constraints were salinity, treatment and time. Scaling = 2. Significance in (a) was tested with 999 permutations. Proportions in (b) refer to total variance. Unique fractions in (f) are adjusted R² values after accounting for the other two constraints. Shared fractions were small or negative and are omitted.

**Table S6.** Pearson correlation coefficients between treatment-mean root traits (24–168 h) and treatment-mean seed cotton yield or NUE in the pot experiment (n = 6)

| **Variable** | **r (Yield)** | **r (NUE)** |
| --- | --- | --- |
| Root dry mass | 0.975*** | −0.783 |
| Root surface area | 0.989*** | −0.702 |
| Urease activity | 0.575 | −0.907* |
| GS | 0.773 | −0.917* |
| GS/GDH ratio | 0.62 | −0.414 |
| GOGAT | 0.709 | −0.912* |
| Root urea content | 0.702 | −0.702 |
| Root NH₄⁺ content | 0.69 | −0.978*** |
| TOC secretion rate | 0.478 | −0.888* |
| Total organic acids | 0.524 | −0.900* |
| Total sugars | 0.471 | −0.871* |

Note: Coefficients are based on six treatment × salinity means. Root traits are means over 24–168 h after the fifth fertilization. Yield and NUE are means of three harvest pots. Root and yield data were not taken from the same plants. *, P < 0.05; ***, P < 0.001.

**Table S7**. Analysis of variance for salinity × treatment effects in the pot experiment and year × salinity × treatment effects in the field trial

(a) Pot experiment (two-way ANOVA: salinity × treatment; n = 3)

| Trait | Salinity | Treatment | Salinity × Treatment |
| --- | --- | --- | --- |
| Total root length | < 0.001 *** | < 0.001 *** | 0.050 * |
| Root surface area | < 0.001 *** | < 0.001 *** | 0.012 * |
| Root volume | < 0.001 *** | < 0.001 *** | 0.006 ** |
| Root dry mass | < 0.001 *** | < 0.001 *** | 0.891 ns |
| Seed cotton yield | < 0.001 *** | < 0.001 *** | 0.046 * |
| Total plant N | < 0.001 *** | < 0.001 *** | 0.032 * |
| NUE | < 0.001 *** | < 0.001 *** | 0.001 *** |

(b) Field trial (three-way ANOVA: year × salinity × treatment; n = 3 per year)

| Trait | Year | Salinity | Treatment | Salinity × Treatment |
| --- | --- | --- | --- | --- |
| Seed cotton yield | 0.122 ns | < 0.001 *** | < 0.001 *** | 0.055 ns |
| Root dry mass | 0.070 ns | < 0.001 *** | < 0.001 *** | 0.620 ns |
| Root urease activity | 0.095 ns | 0.006 ** | < 0.001 *** | 0.413 ns |
| Root urea content | 0.026 * | 0.999 ns | < 0.001 *** | 0.151 ns |
| PFP | 0.225 ns | < 0.001 *** | 0.042 * | 0.970 ns |
| AE | 0.486 ns | < 0.001 *** | < 0.001 *** | 0.907 ns |

(c) Salinity × treatment restricted to U and UN

| Trait | Experiment | Salinity × Treatment |
| --- | --- | --- |
| Total root length | Pot | 0.362 ns |
| Root surface area | Pot | 0.904 ns |
| Root volume | Pot | 0.464 ns |
| Root dry mass | Pot | 0.992 ns |
| Seed cotton yield | Pot | 0.727 ns |
| Total plant N | Pot | 0.761 ns |
| NUE | Pot | 0.024 * |
| Seed cotton yield | Field | 0.970 ns |
| Root dry mass | Field | 0.778 ns |
| Root urease activity | Field | 0.254 ns |
| Root urea content | Field | 0.134 ns |
| PFP | Field | 0.970 ns |
| AE | Field | 0.907 ns |

Notes: Values are P values from Type II ANOVA. CK, no nitrogen; U, urea; UN, urea + NBPT. NUE, nitrogen use efficiency (pot experiment only). PFP, partial factor productivity of N; AE, agronomic efficiency of N (field trial; U and UN only). Pot traits in (a) correspond to Fig. 1 (72 h after the fifth fertilization) and Fig. 5. Field traits in (b) correspond to Figs. 6 and 7. Year × salinity, year × treatment and the three-way interaction in the field trial were not significant (*P* > 0.40). Panel (c) tests whether the UN versus U contrast differed between salinity levels. * *P* < 0.05; ** *P* < 0.01; *** *P* < 0.001; ns, not significant.

**Fig. S1.** GS/GDH ratio (mean values across 24–168 h after the fifth fertilization). Different asterisks indicate significant differences among treatments within the same salinity level (*P < 0.05; **P < 0.01; ***P < 0.001). CK, no nitrogen; U, urea; UN, urea + NBPT; LS, low salinity; MS, moderate salinity.
